# Benign prostatic hyperplasia complicated with T1DM can be alleviated by treadmill exercise—evidences revealed by the rat model

**DOI:** 10.1186/s12894-015-0104-8

**Published:** 2015-11-17

**Authors:** Kuan-Chou Chen, Shian-Ying Sung, Yi-Ting Lin, Chiu-Lan Hsieh, Kun-Hung Shen, Chiung-Chi Peng, Robert Y. Peng

**Affiliations:** Department of Urology, Shuang Ho Hospital, Taipei Medical University, 291 Zhongzheng Rd.,, Zhonghe, Taipei 23561 Taiwan; Department of Urology, School of Medicine, College of Medicine, Taipei Medical University, 250 Wu-Shing St., Taipei, 11031 Taiwan; The Ph. D. Program for Translational Medicine, College of Medical Science and Technology, Taipei Medical University, Taipei, Taiwan; Department of Urology, St. Joseph’s Hospital, 74, Sinsheng Road, Huwei County, Yunlin Hsien 632 Taiwan; Research Institute of Biotechnology, Hungkuang University, 34 Chung-Chie Rd., Shalu County, Taichung Hsien 43302 Taiwan; Graduate Institute of Biotechnology, Changhua University of Education, 1 Jin-De Rd., Changhua, 50007 Taiwan; Division of Urology, Department of Surgery, Chi-Mei Medical Center, 901 Chung Hwa Road, Yung Kang City, Tainan 701 Taiwan; Graduate Institute of Clinical Medicine, College of Medicine, Taipei Medical University, 250 Wu-Shing St., Xin-Yi District, Taipei 110 Taiwan

**Keywords:** Exercise, BPH, T1DM, Insulin, Androgen, Nitric oxide (NO)

## Abstract

**Background:**

Both benign prostatic hyperplasia (BPH) and Type-1 diabetes mellitus (T1DM) share similar epidemiologic features and are all associated with the insulin-like growth factor (IGF)-mediated hormonal imbalance. The purpose of this study is to understand whether exercise (EX) could alleviate DM and DM + BPH.

**Methods:**

Sprague-Dawley rats were divided into eight groups: normal control, EX, BPH, BPH + EX, DM, DM + EX, BPH + DM, and BPH + DM + EX. T1DM was induced by intraperitoneal (ip) injection of streptozotocin (65 mg/kg) in Week 2, and BPH was induced by successive ip injections of Sustanon® (testosterone, 3.5 mg/head) plus estradiol (0.1 mg/head) from Week 3 to Week 9. Treadmill exercise training (20 m/min, 60 min per time) was performed three times per week for 6 weeks.

**Results:**

In BPH + EX, EX maintained at a constant body weight (BW); and suppressed stromal layer thickening, collagen deposition, blood glucose (BG), levels of testosterone (Ts), 5α-reductase(5αRd), dihydrotestosterone (DHT), androgen receptor (AR), serum hydrogen peroxide, TBARs, and interleukin-6 (IL-6). EX recovered testes size and substantially increased nitric oxide (NO) levels. In DM + EX group, EX decreased BW, PW, nuclear proliferation, inflammatory cell aggregation, collagen deposition, and BG. As contrast, EX upregulated insulin, IGF, Ts, NO, 5αRd, AR, and DHT, and substantially reduced PSA. In BPH + DM + EX, EX maintained BW at a subnormal level, slightly suppressed prostate stromal inflammation, collagen deposition, and BG, moderately restored sIn and IGF. Although failed to suppress Ts, EX highly upregulated 5αRd and suppressed DHT and AR, together with highly upregulated NO resulting in substantially reduced PSA.

**Conclusion:**

EX, by remodeling androgen and NO expressions, can effectively alleviate BPH, DM, and BPH + DM.

## Background

Benign prostatic hyperplasia (BPH) is the most common benign tumor in men. Epidemiological data have indicated that BPH may be associated with the metabolic syndrome (MetS) [1] which can substantially increase the risk of BPH and low urinary tract symptoms (LUTS) [2].

When complicated with diabetes mellitus (DM), the mechanisms that regulate reactive stroma biology in BPH can be altered anatomically, pathologically, and biochemically [3]. Prostatic volume and the anterior-posterior diameter are positively associated with the component number of MetS [1]. The possible pathophysiologic mechanisms needed to explain these relations include an increased sympathetic tone, the alterations in sex steroid hormone expression, and the induction of systemic inflammation and oxidative stress [4]. The levels of insulin-like growth factor (IGF) and IGF-binding proteins (IGFBPs) in prostate tissue and blood are associated with the risk of developing BPH, which also regulate the circulating androgen and growth hormones [2].

Regular exercise (EX) is associated with low levels of interleukin-6 (IL-6), tumor necrosis factor-α (TNF-*α*), and simultaneously, with increases in antiinflammatory substances, such as adiponectin, IL-4, and IL-10 [5]. Hence, moderate EX training can exert antioxidant and antiinflammatory systemic protective effects [5]. Much of the literature supports a clinically relevant, independent, and strong inverse relationship between EX and the development of BPH and LUTS [6–8]. Furthermore, running considerable distances per week may lower the BPH risk, independent of the BMI and diet [9].

EX has been shown to have beneficially improved the Type 2 DM (T2DM) [10, 11] and BPH [9]. Moreover, amounting evidences also have revealed that a close association between BPH and T2DM through a common pathogenic mechanism is possible [12]. Parsons et al. in a chort report indicated that obesity, elevated fasting plasma glucose level, and DM are risk factors for BPH [13]. Previous document even substantially pointed out that diabetic vascular damage may cause hypoxia which in turn may contribute to pathogenesis of BPH [14]. Recently, we have showed EX beneficially alleviated BPH [7], however, the documented effect of EX on patients concomitantly affiliated with BPH plus Type 1 DM (BPH + T1DM) is still lacking. We hypothesized that EX could be beneficial to BHP + T1DM subjects. In this present study we developed a BPH + DM rat model to verify whether EX could improve such a metabolic syndrome.

## Methods

### Chemicals

T-Pro Western Blot Stripping Reagent was obtained from BioPioneer (San Diego, CA, USA); streptozotocin (STZ), Sirius Red, bovine serum albumin (BSA), and sodium dodecyl sulfate-polyacrylamide were purchased from Sigma-Aldrich (St. Louis, MO, USA); Coomassie Brillant Blue R-250, Coomassie Brilliant Blue-G, glycine, and Tris base were obtained from US Biological (USA). Bis-acrylamide solution was purchased from Serva (Germany). Sustanon® was provided by the Schering-Plough Company (Kenilworth, NJ, USA) which is an injectable testosterone medication containing four testosterone esters at concentrations: 30 mg/mL of testosterone propionate, 60 mg/mL of testosterone phenylpropionate, 60 mg/mL of testosterone isocaproate, and 100 mg/mL of testosterone decanoate. The overall androgenic potency per mL of Sustanon® is equivalent to 176 mg of testosterone. Tris (hydroxymethyl) aminomethane hydrochloride (Tris-HCl) and hydrogen peroxide were purchased from Panreac (Spain). PageRuler™ Prestained Protein Ladder was supplied by Fermentas (Canada). TEMED, ammonium persulfate (APS), and mineral oil were products of Bio-Rad (USA).

The sources of various kits were: Rat insulin ELISA kit (Mercodia, Sweden), AssayMax mouse insulin-like growth factor-1 (IGF-1 ELISA Kit; AssayPro, USA), rat Interleukin-6 (IL-6) ELISA kit (PeproTech, USA). TBARS ELISA kit (Cayman Chemical, USA), hydrogen peroxide (H_2_O_2_) assay kit (BioVision, USA) testosterone EIA (Cayman Chemical, USA), and dihydrotestosterone ELISA kit (Alpha Diagnostic, USA).

While the suppliers of antibodies were: antirabbit IgG (eBioscience, USA), antimouse IgG (Jackson ImmunoResearch, USA), β-actin antibody (Novus Biologicals, USA), antigoat IgG, 5α-reductase antibody, androgen receptor antibody (Santa Cruz Co., USA), and prostatic-specific antigen (PSA) antibody (Bioss, Scotland).

### Animals

This experiment was approved by the Institutional Animal Care and Ethics Committee of Taipei Medical University (Taipei, Taiwan), and adhered to the animal care standards of the American College of Sports Medicine. In brief, 64 male Sprague-Dawley rats, aged 6 weeks, weighing 250–265 g were purchased from Biolasco Co. (Taipei, Taiwan). The rats were housed in an animal room conditioned at 24 ± 2 °C, RH 70–75 %, with a 12 h/12 h light/night cycle. The access of water and chow was ad libitum. The animals were acclimated in the animal room during the first week and then divided into eight groups, with eight rats in each group: Group 1, normal control; Group 2, BPH control; Group 3, DM control; Group 4, BPH + DM; Group 5, EX control; Group 6, BPH + EX; Group 7, DM + EX; and Group 8, BPH + DM + EX. The animals were separately caged, with 2 rats in each cage. In Week 2, DM groups were induced with a single intraperitoneal (ip) injection of streptozotocin (65 mg/kg) The BPH groups were induced in Week 3 by daily ip injection with Sustanon® (testosterone, 3.5 mg/head) and estradiol (0.1 mg/head), consecutively for 8 weeks. Exercise training was conducted from Week 12 until Week 17 on a rat exercise treadmill (Fortelice, International Co., Ltd., Taiwan). according to the program: rats were allowed to sprint at 20 m/min, 60 min per time, three courses per week, this program was continued successively for a total period of 6 weeks.

### Blood collection and analysis of the lipid profiles

The control biochemical data were established at the end of the first week before experiment. The control data of the DM-control was established 1 week after STZ-induction, and those of the BPH-control group was collected 8 weeks after Sustanon®-induction. Blood collection was performed at the end of Week 2 and Week 17. In Week 17, the rats were bled from the abdominal arteriole immediately before euthanized with CO_2_ anesthesia. The blood obtained was centrifuged at 4 °C at 3000 × g for 10 min using a freezer-type centrifuge (1580 MGR, Gyrozen, Korea), and the serum high-density lipoprotein (HDL), serum low-density lipoprotein (LDL), serum cholesterol (CHOL), and triglyceride (TG) were determined using respective kits by following the manufacturer’s instructions.

### Collection of tissue specimens

After euthanized, the prostates with seminiferous vesicles and testis were excised, photoed and weighed. Half of each organ was immersed in a 10 % formalin fixation solution, and the other half was rapidly immersed into liquid nitrogen, and stored at −80 °C for further use.

### Extraction of proteins

To 200 mg prostate tissues lysis buffer (1.6 mL) was added, homogenized (microquantity-type homogenizer, T10 Basic, IKA, Germany) on ice and left to react for 30 min. The homogenate was centrifuged using the freezer-type centrifuge (1580 MGR) at 12000 × g at 4 °C for 20 min. The supernatant (protein lysate, PLS) was separated and stored at −80 °C for further use.

### Western blot analysis of 5α-reductase

The PLS was assayed for total protein content. To PSL, a two-fold volume of Western sample loading dye (WSLD) solution was loaded. The mixture (named herein PSL-WSLD) was heated in a dry heating bath (100 °C) for 10 min, and treated as follows. PSL-WSLD containing 30 μg of protein was loaded onto 10 % SDS-PAGE and the electrophoresis was conducted in the SDS-PAGE electrophoresis chamber (Mini-Protean Tetra Cell, Bio-Rad, USA), using the SDS-PAGE electrophoresis buffer (running buffer of pH 8.3, containing 25 mM Tri-HCl, 192 mM glycine, 0.1 % SDS, and deionized water to adjust to 1 L) at 75 V for 30 min. The protein spots were electrotransferred onto the PDVF membrane using a Mini Trans-Blot (Bio-Rad, USA) at 4 °C and 75 V for 20 h. The PVDF membrane was removed and marked with the obtained molecular weight. The marked membrane was sliced according to the molecular weight, immersed in a blocking buffer, and agitated at 4 °C overnight. The membrane was rinsed with a TBST solution thrice and left to stand for 10 min. The primary antibodies were applied and left to react for 1 h at ambient temperature, and then rinsed with the TBST solution. The secondary antibodies were applied, and left to react at ambient temperature for 1 h. After rinsed with the TBST solution, enhanced chemiluminescence (ECL) was applied and left to react completely. Protein expression was imaged using a luminescent image analyzer (LAS-4000; Fujifilm, Tokyo, Japan).

### Enzyme linked immunosorbent analysis for determining serum insulin, IGF, TBARS, H_2_O_2_, testosterone, DHT and prostate IL-6

The blood obtained was immediately centrifuged using the freezer-type centrifuge (1580 MGR) at 3000 × g and 4 °C for 10 min. The supernatant serum was separated and stored at −80 °C if not used immediately. The sera were used for determining insulin, IGF, TBARS, H_2_O_2_, testosterone and DHT. Prostatic tissues (100 mg) were minced into chops having size <3 mm^3^ and extensively washed with PBS containing heparin to prevent potential peripheral blood contamination. The mince was incubated with 200 U/mL type I collagenase and 100 mg/mL DNase type I (Sigma Chemical Company, St. Louis, MO) in RPMI 1640 medium plus 10 % fetal calf serum and 6 % penicillin/streptomycin solution (Gibco BRL Life Technologies, Gaithersburg). The tissues were dissociated overnight at 37 °C and used for determination of IL-6. The following protocol for assay was performed following the instructions given by the manufacturers.

### Pathological examination and Sirius Red staining

After CO_2_-euthanized, prostate, testes, seminal vesicle, bladder, pancreas, kidneys, heart, liver, and muscles were excised, photoed and weighed. Half of each organ was fixed in 10 % formalin, paraffin-embedded, and sliced with a microtome. The specimens were forwarded to the National Laboratory Animal Center (NLAC, Taipei) to receive pathological examination.

The paraffin-embedded specimens were dewaxed with xylene, and rehydrated successively with gradient ethanol solutions (100, 95, 80, and 70 %). These specimens were first stained with the Fouchet dying agent to attain a clear contrasting background, then with Weigert’s haematoxylin to stain the nuclei (to a bluish-black color), and finally with Sirius Red to stain the collagen (to red). The semifinished specimens were immediately dehydrated, mounted, and examined using an optical microscope (BX41M-ESD, Olympus, Japan).

### Immunohistochemical stain for androgen receptor (AR) and prostatic specific antigen (PSA)

The paraffin-embedded tissue specimens were placed in an incubator held at 37 °C overnight, immersed in xylene for 10 min to remove the residual embedding paraffin, and successively rehydrated with gradient ethanol solutions (100, 95, 80, and 70 %). Citric acid (10 mM, pH 6.0) was added to the rehydrated specimens. After 15 min, the specimens were treated with 3 % H_2_O_2_ for 15 min and then rinsed twice with PBS. The primary antibodies were applied and left to react for 2 h. After rinsed twice with PBS, the secondary antibodies were applied, left to react for 30 min, and peroxidase-conjugated streptavidin was added to react for 1 h and the specimens were rinsed with PBS twice. Finally, the specimens were reacted with the coloring agent diaminobenzidine (DAB) for 30 min, rinsed twice with PBS, dehydrated with gradient ethanol solutions, and mounted.

### Determination of NO

Griess reagent (20 μL) was added to 20 μL of serum (or tissue homogenate) and mixed well. Double-distilled water (160 μL) was added to the mixture to make up to a total volume 200 μL. The optical density was read at 550 nm against the blank. A calibration curve was established using 20 μL of a standard sodium nitrite solution, similarly treated with 20 μL of Griess reagent and 160 μL of double-distilled water, and finally the optical density was read at 550 nm. The nitric oxide content of the samples was calculated from the reference curve.

### Statistical analysis

Data obtained in the same group were analyzed with Duncan's multiple range test, using the Statistical Analysis System software (SAS 9.0). Data were expressed as mean ± SD. Different letters indicated significant differences at a confidence level of *p* < 0.05.

## Results

### Body weight variation was affected by the treatments

**Fig. 1:**
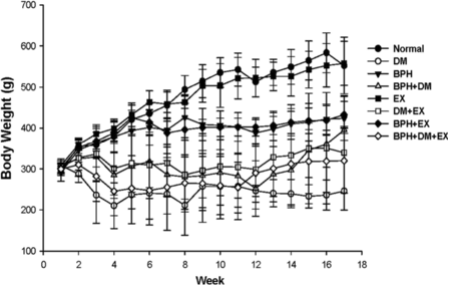
Body weight variation affected by T1DM, BPH, and exercise intervention. Data are presented as mean ± SD (*n* =8)

After BPH induction, the body weight of the normal control and EX control groups steadily increased from Week 3 until Week 17, reaching 552.5 ± 68.6 g and 557.8 ± 53.4 g, respectively. The body weights of the BPH and BPH + EX groups increased for the initial 2 weeks until Week 5 to 423.7 ± 53.4 g and 433.0 ± 32.1 g, respectively, and then remained unchanged until Week 17. In the DM group, a body weight of approximately 246.0 ± 40.0 g almost remained unchanged all the way until Week 17. The body weight of the DM + EX group increased steadily to 339.5 ± 14.01 g after the EX intervention. By contrast, the BPH + DM and BPH + DM + EX groups rapidly gained weight to 395.7 ± 8.6 g and 320.0 ± 79.4 g, respectively, in Week 17 (Fig. 1).

### Testes weights

**Fig. 2:**
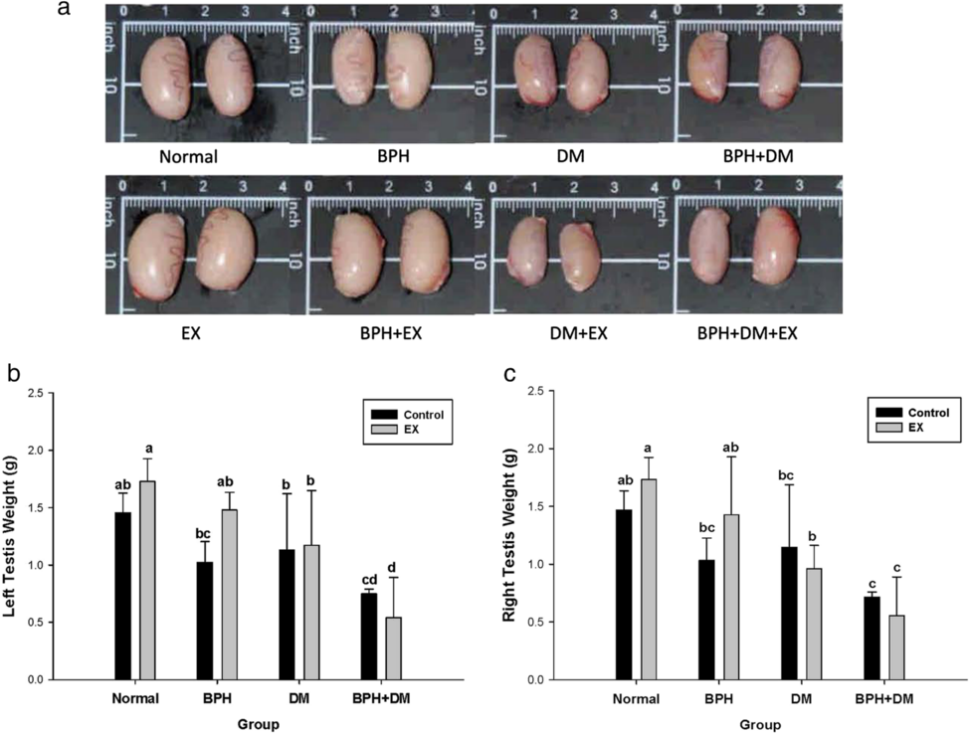
The morphology and size of testis affected by T1DM, BPH, and exercise intervention. Dimensions are indicated by the real length in cm and inch (**a**). The left testes weight are indicated as mean ± SD (**b**). The right testes weight are indicated as mean ± SD (**c**). The different symbols in lower case indicate significantly different from each other (*p* < 0.05). The symbol ‘a’ denotes the highest data, ‘b’ the next, and so on

Figure 2a-c reveals the testis of BPH, DM, BPH + DM, DM + EX, and BPH + DM + EX were all much smaller in size compared to the normal. Ex greatly increased the testes size in BPH group but not in groups DM, BPH + DM, and BPH + DM + EX (Fig. 2b, c).

### Apparent prostatic size and weight were affected by the treatments

**Fig. 3:**
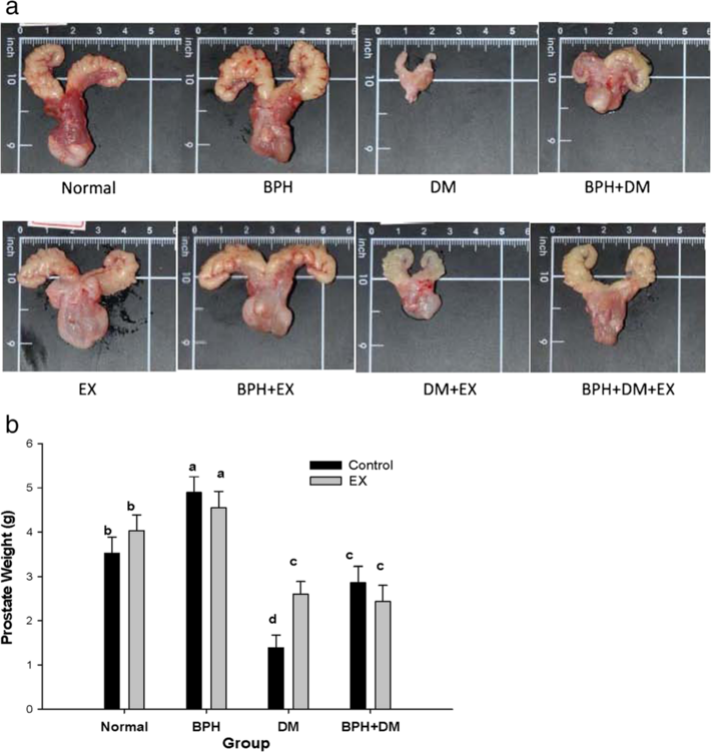
Prostatic morphology (**a**) and prostatic weight (**b**) affected by T1DM, BPH, and exercise intervention. Dimensions are indicated by the real length in cm and inch (**a**). The prostatic weight are indicated as mean ± SD. The different symbols in lower case indicate significantly different from each other (*p* < 0.05). The symbol ‘a’ denotes the highest data, ‘b’ the next, and so on

The mean prostatic weights of the normal control, EX control, BPH control, BPH + EX, DM control, DM + EX, BPH + DM, and BPH + DM + EX groups were 3.52 ± 0.76 g, 4.03 ± 0.76 g, 4.90 ± 0.65 g, 4.55 ± 0.80 g, 1.38 ± 0.89 g, 2.60 ± 1.09 g, 2.86 ± 0.67 g, and 2.43 ± 1.17 g, respectively (Fig. 3b). Compared with the normal prostatic size, the EX control exhibited a slightly enlarged prostate. The morphology and weight of the prostate in the EX and BPH + EX groups was comparable (Fig. 3a and b), but a substantial prostatic weight difference was observed between the normal and BPH groups (*p* < 0.05) (Fig. 3b). The prostate in the DM groups was severely shrunken (Fig. 3a and b). In the BPH + DM group, the extent of shrinkage was much less severe than that in the DM group. EX increased the prostatic size and weight in the DM + EX group (*p* < 0.05), but not in the BPH + DM + EX group (Fig. 3b).

### Lipid profile

**Fig. 4:**
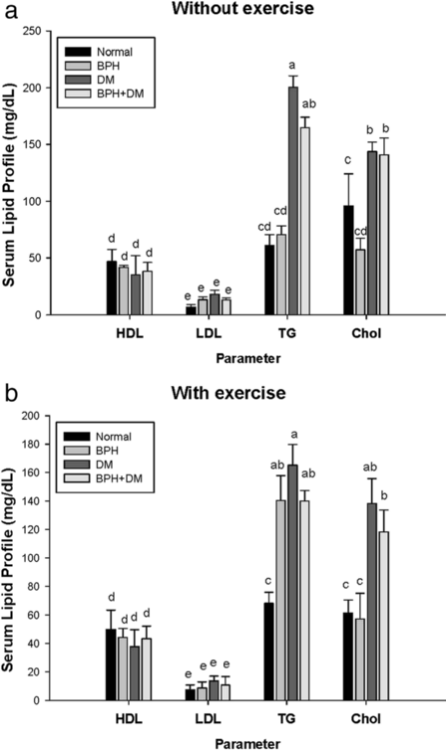
The level of serum lipid profile without (**a**) and with (**b**) exercise. The different symbols in lower case indicate significantly different from each other (*p* < 0.05). The symbol ‘a’ denotes the highest data, ‘b’ the next, and so on

The level of HDL for the control, BPH, DM, and BPH + DM value without EX were 46.8 ± 10.8 mg/dL, 41.7 ± 1.7 mg/dL, 35.4 ± 16 mg/dL, and 38.5 ± 7.6 mg/dL, respectively. While EX altered the corresponding values to 49.9 ± 13.6 mg/dL, 44.3 ± 6.3 mg/dL, 37.8 ± 11.8.mg/dL, and 43.3 ± 8.9 mg/dL, respectively (Fig. 4a-b).

Likely, without EX intervention, the serum LDL level of BPH, DM, and BPH + DM were substantially raised to 13.4 ± 0.5 mg/dL, 18.2 ± 0.5 mg/dL, and 13.1 ± 1.8 mg/dL, respectively compared to the control 6.8 ± 0.8 mg/dL (Fig. 4a). EX correspondingly alleviated the values to 8.5 ± 4.4 mg/dL, 13.6 ± 3.3 mg/dL, and 10.6 ± 6.1 mg/dL, respectively, comparing with the EX control 7.3 ± 3.4 mg/dL (Fig. 4b).

Moreover, without the EX intervention, the ratio LDL/HDL revealed to be 0.15, 0.32, 0.51, and 0.34 for groups normal, BPH, DM, and BPH + DM. As contrast, EX intervention greatly improved these ratio to 0.15, 0.19, 0.36, and 0.24, respectively.

Without EX intervention, DM and BPH + DM highly raised the serum TG levels to 200.5 ± 10.1 mg/dL, and 164.7 ± 9.4 mg/dL compared to the control 61.1 ± 9.6 mg/dL (note: The normal gross range is 26–145 mg/dL) (Fig. 4a). Although BPH had slightly raised this value to 70.7 ± 7.7 mg/dL, it still fell in normal gross range. Similarly, without EX intervention DM and DM + BPH elevated the serum CHOL level to 143.8 ± 8.2 mg/dL, and 140.7 ± 15.0 mg/dL. EX intervention slightly suppressed the CHOL levels in DM and BPH + DM to 138.4 ± 17.4 mg/dL, and 118.3 ± 15.4 mg/dL respectively, while BPH group showed 57.2 ± 18.1 mg/dL compared to the EX control 61.3 ± 9.3 mg/dL (Fig. 4b).

### Hematoxylin-eosin staining

**Fig. 5:**
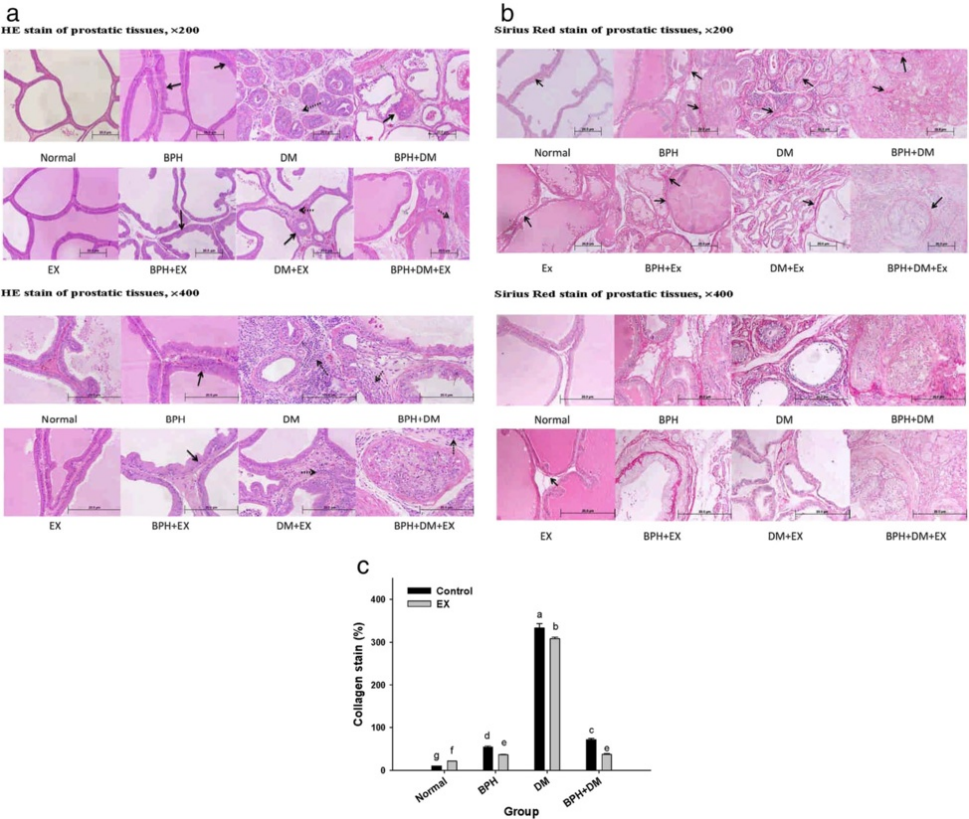
Hematoxylin-eosin stain (**a**), Sirius Red stain (**b**), and collagen dposition (**c**) of prostatic tissues affected by different treatments. (in (**a**) and (**b**), magnification: upper × 200, lower panel × 400). Different symbols in lower case in the Fig. 5c indicate significantly different from each other (*p* < 0.05). The symbol ‘a’ denotes the highest data, ‘b’ the next, and so on

The prostatic tissues of the BPH group exhibited the manifestation of epithelial hyperplasia (epithelial cells piling-up formation)(indicated by solid arrows), as well as slight interstitial leukocytic infiltration (Fig. 5a and b). The prostate of DM rats revealed epithelial hyperplasia, moderate to severe inflammation, moderate lymphocytic infiltration (indicated by dotted arrows), and slight to moderately severe acinar atrophy (Fig. 5a and b). In BPH + DM rats, a high degree of hyperplasia and severe inflammation were observed. EX alleviated most of these pathological events (Fig. 4a and b; see BPH + EX, DM + EX, and BPH + DM + EX).

### Sirius Red staining

Sirius Red stains the collagen to red, in essence underlying severe collagen deposition with inflammation of interstitial tissues. In the BPH groups, collagen deposition substantially increased to 54.32 ± 2.19 %, which was further enhanced to 333.46 ± 10.35 % in the DM groups, compared with the normal control value (10.25 ± 0.28 %). EX substantially reduced the collagen deposition to 36.37 ± 0.74 % and 308 ± 3.56 % in the BPH and DM groups, respectively (*p* < 0.01) (Fig. 5c). Remarkably, collagen deposition in the BPH + DM group, whose control exhibited an initial deposition level of 71.62 ± 2.78 %, was also substantially suppressed by EX to 37.24 ± 2.20 % (*p* < 0.05) (Fig. 5c).

### Blood glucose levels were affected by BPH, DM, and EX

**Fig. 6:**
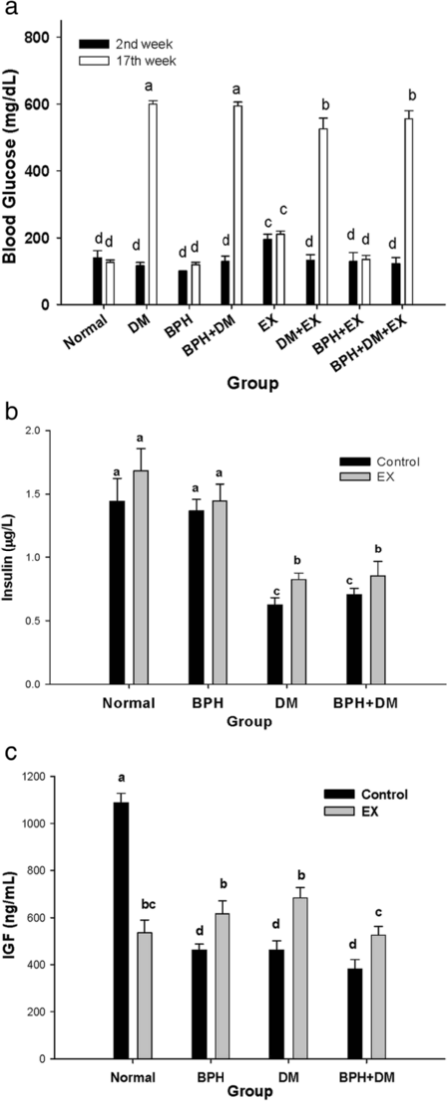
Levels of blood glucose (**a**), insulin (**b**), and insulin growth factor (**c**) affected by T1DM, BPH, and exercise intervention. Data are expressed as means ± SD. Different symbols in lower case indicate significantly different from each other . The symbol ‘a’ denotes the highest data, ‘b’ the next, and so on

In Week 17, the mean blood glucose (BG) levels in the normal, BPH, EX, and BPH + EX groups were 127.0 ± 7.1 mg/dL, 119.7 ± 7.0 mg/dL, 210.5 ± 9.8 mg/dL, and 135.0 ± 12.5 mg/dL, respectively(*p* < 0.05). In the DM groups (DM, BPH + DM, DM + EX, and BPH + DM + EX), the corresponding values were 600.0 ± 10.1 mg/dL, 594.1 ± 12.0 mg/dL, 525.8 ± 31.7 mg/dL, and 555.4 ± 24.9 mg/dL, respectively (*p* < 0.01) (Fig. 6a). Thus, DM prominently increased 4.14–4.72 folds the serum glucose levels in the DM groups. In the DM + EX and BPH + DM groups, the blood sugar levels were substantially, yet only slightly, decreased (Fig. 6a).

### Serum insulin levels in the BPH, DM, and EX groups

The insulin levels in the control and BPH groups were comparable, ranging from 1.45 ± 0.13 μg/L to 1.68 ± 0.18 μg/L regardless of EX (Fig. 6b). In the DM group the insulin level was lowered to only 0.62 ± 0.06 μg/L, which was substantially improved by EX to 0.82 ± 0.05 μg/L (*p* < 0.05). A similar trend was observed in the BPH + DM group.

### Insulin-like growth factor was affected by BPH, DM, and EX

The IGF in the normal control was 1087.3 ± 41.0 ng/mL, EX reduced this level to 535.2 ± 54.9 ng/mL (*p* < 0.01). In the BPH, DM, and BPH + DM groups, EX substantially raised the IGF, compared with each corresponding control groups (*p* < 0.05) (Fig. 6c).

### Androgen-related biochemical parameters affected by BPH, DM, and EX

#### Testosterone

**Fig. 7:**
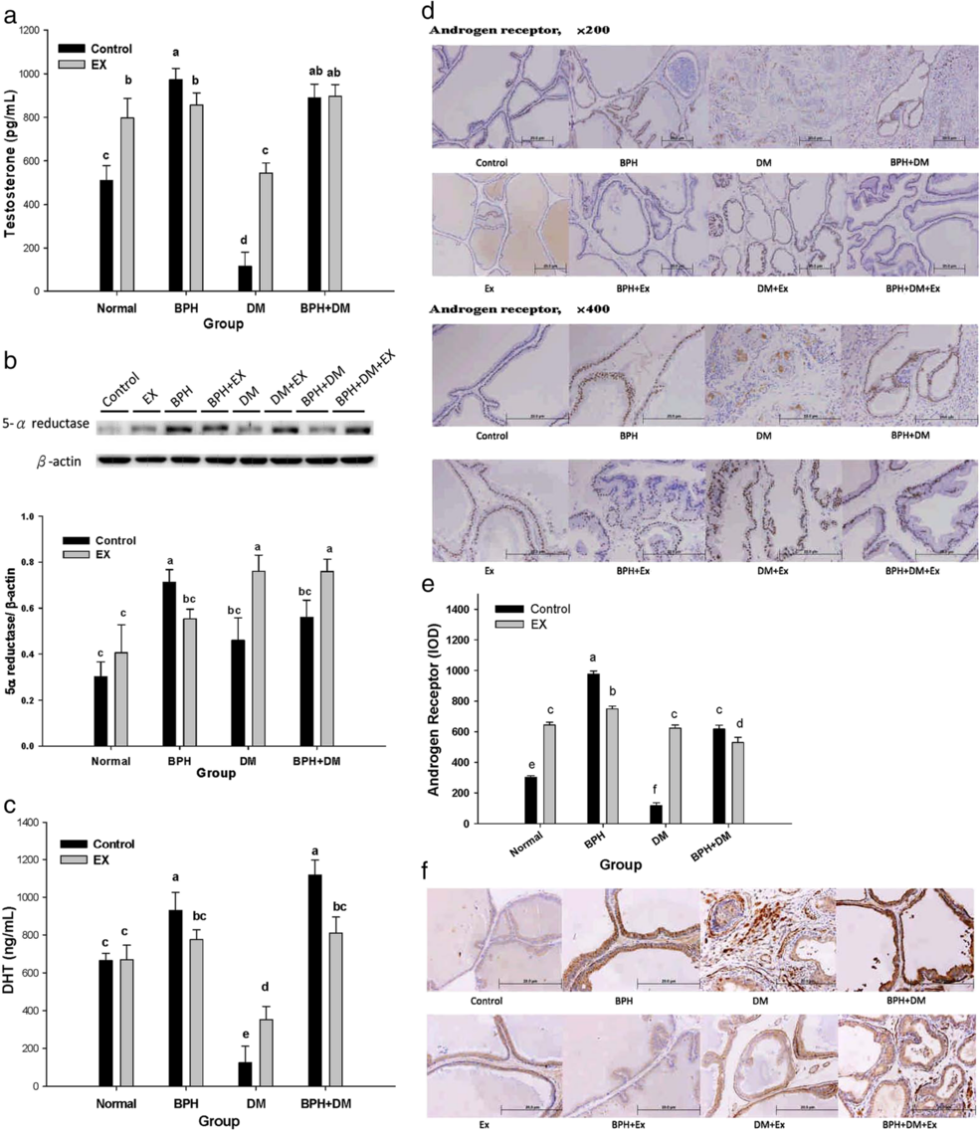
Androgen-related variables affected by T1DM, BPH, and exercise intervention. **a** Testosterone, **b** 5α-reductase, **c** DHT, **d** AR (upper, ×200; lower, ×400), **e** quantitatification of AR, **f** PSA (magnification, ×200). Data are expressed as mean ± SD (*n* = 8). Different symbols indicates statistically different (*p* < 0.05). The different symbols in lower case indicate significantly different from each other. The symbol ‘a’ denotes the highest data, ‘b’ the next, and so on

EX considerably increased the serum testosterone levels to 797.2 ± 89.6 pg/mL, compared with the control value of 508.6 ± 69.5 pg/mL. BPH increased this concentration to 972.6 ± 52.4 pg/mL (*p* < 0.01), whereas EX substantially lowered it to 856.8 ± 55.5 pg/mL (*p* < 0.05). By contrast, the testosterone level was highly suppressed in the DM control group to 115.5 ± 66.1 pg/mL, and EX restored its level to 543.7 ± 46.6 pg/mL (*p* < 0.01), being comparable to that in the normal control. In the BPH + DM and BPH + DM + EX groups, the testosterone levels were increased to 889.3 ± 62.9 pg/mL and 896.1 ± 54.6 pg/mL, respectively (Fig. 7a).

#### 5α-Reductase

In contrast with the testosterone profile, 5α-reductase exhibited a similar profile in all groups. The relative data for the normal control, EX control, BPH control, BPH + EX, DM control, DM + EX, BPH + DM, and BPH + DM + EX groups were 0.30 ± 0.07, 0.41 ± 0.12, 0.71 ± 0.05, 0.55 ± 0.04, 0.46 ± 0.10, 0.76 ± 0.07, 0.56 ± 0.07, and 0.76 ± 0.05 folds contrasting to the reference β-actin, respectively (*p* < 0.05) (Fig. 7b).

EX upregulated the 5α-reductase levels in the normal group, although the change was negligible (Fig. 7b). However, 5α-reductase activity was substantially upregulated in the DM + EX and BPH + DM + EX groups. By contrast, 5α-reductase activity was substantially inhibited in the BPH + EX group (Fig. 7b).

#### Dihydrotestosterone

The normal, EX and DM controls exhibited dihydrotestosterone (DHT) levels of 665.9 ± 37.7 pg/mL, 669.8 ± 76.7 pg/mL, and 122.8 ± 88.4 pg/mL, respectively (*p* < 0.01). EX did not alter the normal DHT level, whereas EX substantially raised, yet incompletely restored, the DHT level in the DM group to 351.96 ± 70.34 ng/mL (*p* < 0.01) (Fig. 7c). Because of the administration of testosterone (Sustanon®), the DHT of all BPH groups was raised to levels higher than those in the normal and DM groups (Fig. 7c). EX effectively suppressed the DHT levels in the BPH + EX and BPH + DM + EX groups. Conversely, EX increased the DHT levels in DM + EX rats (Fig. 7c).

#### Androgen receptor and prostate-specific antigen

The androgen receptor (AR) that was once highly expressed in BPH was substantially suppressed by EX. By contrast, AR in the DM control appeared to be more diffusively spreading due to the degenerative destruction of the tissues (Fig. 7d and e). EX effectively upregulated AR in the normal and DM groups (*p* < 0.01), but apparently lowered AR in the BPH and BPH + DM groups (*p* < 0.05) (Fig. 7d (×400) and e). In parallel, the PSA level in the normal control was slightly upregulated, and that of BPH and BPH + DM groups were significantly lowered (Fig. 7f). Interestingly, the PSA of DM rats was more apparently localized and dowregulated by the EX intervention (Fig. 7f)

### Pro-inflammatory factors affected by BPH, DM, and EX

#### Serum hydrogen peroxide

**Fig. 8:**
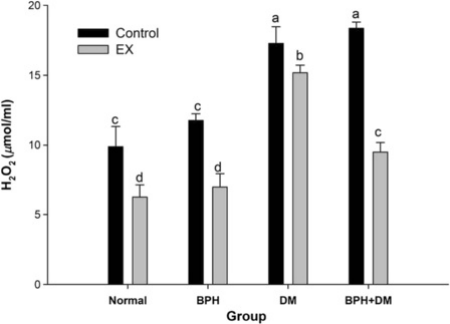
Levels of serum hydrogen peroxide affected by T1DM, BPH, and exercise intervention. Different symbols indicates statistically different (*p* < 0.05). The different symbols in lower case indicate significantly different from each other. The symbol ‘a’ denotes the highest data, ‘b’ the next, and so on

The serum hydrogen peroxide levels were highly stimulated to 17.27 ± 1.19 and 18.34 ± 0.45 μmol/mL respectively in the control groups of DM and BPH + DM compared to the normal 9.88 ± 1.45 and the BPH control 11.75 ± 0.48 μmol/mL (*p* < 0.05) (Fig. 7). EX substantially suppressed the serum hydrogen peroxide levels to 6.25 ± 0.87, 6.96 ± 0.96, 15.18 ± 0.54, and 9.49 ± 0.68 μmol/mL, respectively for groups normal, BPH, DM, and BPH + DM (*p* < 0.05) (Fig. 8).

#### Serum TBARS

**Fig. 9:**
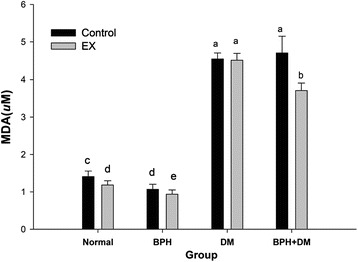
Levels of serum TBARs affected by T1DM, BPH, and exercise intervention. Different symbols indicates statistically different (*p* < 0.05). The different symbols in lower case indicate significantly different from each other. The symbol ‘a’ denotes the highest data, ‘b’ the next, and so on

The serum TBARs levels were slightly suppressed in groups normal and BPH compare to each individual control. In the DM and BPH + DM controls, the serum TBARS were highly stimulated to levels 4.54 ± 0.16 μM and 4.7 ± 0.45 μM, respectively (Fig. 9). Although EX completely failed to improve the TBRAs level in DM group, EX showed slight yet significant suppressive effect on the TBARS level in the BPH + DM rats (*p* < 0.05) (Fig. 9).

#### Prostate tissue IL-6

**Fig. 10:**
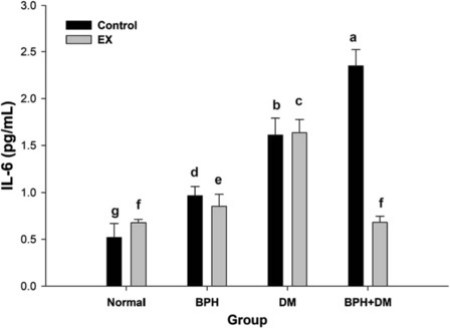
Levels of prostatic IL-6 affected by T1DM, BPH, and exercise intervention. Different symbols indicates statistically different (*p* < 0.05). The different symbols in lower case indicate significantly different from each other. The symbol ‘a’ denotes the highest data, ‘b’ the next, and so on

The IL-6 levels of each controls were all raised in groups BPH, DM, and BPH + DM. The levels reached 0.96 ± 0.1, 1.61 ± 0.18, and 2.35 ± 0.17 pg/mL, respectively (Fig. 10). EX slightly raised the IL-6 level to 0.68 ± 0.04 pg/mL compared to the normal. Conversely, EX slightly but significantly suppressed the IL-6 levels in groups BPH and DM. Astonishingly, EX effectively alleviated the IL-6 level from 2.4 ± 0.4 pg/mL down to 0.68 ± 0.06 pg/mL in group BPH + DM (*p* < 0.01) (Fig. 10).

#### Serum nitric oxide

**Fig. 11:**
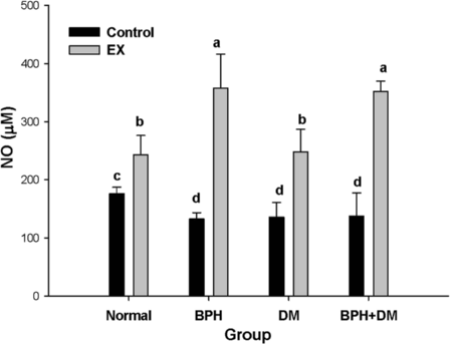
Serum nitric oxide affected by T1DM, BPH, and exercise intervention. The different symbols in lower case in the figure indicate significantly different from different groups (*p* < 0.05). The symbol ‘a’ denotes the highest data, ‘b’ the next, and so on

The serum nitric oxide (NO) was upregulated by EX in all experimental groups, particularly in the BPH group. The serum NO levels were 175.5 ± 11.2 μM, 243.5 ± 33.1 μM, 132.6 ± 10.5 μM, 357.6 ± 58.7 μM, 135.6 ± 25.0 μM, 248.7 ± 38.4 μM, 137.1 ± 40.0 μM, and 352.3 ± 17.9 μM, respectively for the normal control, EX control, BPH control, BPH + EX, DM control, DM + EX, BPH + DM, and BPH + DM + EX groups (*p* < 0.05) (Fig. 11).

## Discussion

### DM severely reduced the prostatic weight, whereas EX increased that of DM group but not that of BPH + DM group

Pathologically, BPH is characterized by hyperplastic epithelial, stromal growth [15] and tissue remodeling in the aging prostate [16], which was consistent with our findings (Fig. 5a and b). Stromal-epithelial interaction plays a critical role in the development and growth of the prostate gland and BPH [2]. Ikeda et al. indicated that DM caused a substantial reduction in prostatic weight and serum testosterone levels in rats [17]. Similar results were also reported by Porto et al. [18]. The prostate weight was substantially increased in the BPH control, compared with the normal control. EX did not substantially affect the prostatic weight (Fig. 3b). Conversely, DM remarkably reduced the prostatic weight, and EX effectively inhibited the reduction in prostatic weight (Fig. 3b).

Literature elsewhere also indicated that BPH exhibits manifestations of hypoxia and chronic inflammation [19]. We showed that EX slightly improved hyperplasia and inflammation of the dorsolateral lobe (NLAC report, not shown here) (Fig. 5a and b), and DM caused epithelial hyperplasia inflammation, lymphocytic infiltration, and acinar atrophy (NLAC report, not shown here). As contrast, EX alleviated the chronic inflammation of the DM + EX group (Fig. 5a and b).

Here some controversial arguments may arise. One may claim about the T1DM model to be characterized by some important histopathological alterations that are typical of the initial phase of BPH, including inflammation, tissue remodeling and increased stromal proliferation and more importantly, which is associated to a condition of hypogonadism as already reported by others [20] and pointed out that such a condition of hypogonadism is also associated with significant prostate alterations.

However, there in fact exists a big discrepancy between the DM model and the BPH model. As cited by Zhang et al. [20], the DM animal models could be elicited by two techniques, one by alloxan, and the other by streptozotocin (STZ). Indeed, in the DM model, a state of hypogonadism would have occurred, and supplement with testosterone definitely would be beneficial [20]. However, we were creating a complicate animal model that is DM + BPH. DM model was induced at week 2 by STZ, and the BPH model was then induced one week later using T + E. Recently, the pathological etiology and clinical findings for BPH all are pointing to the hypertestosteronemia, dyslipidemia, and oxidative stress. In our experiment, we really have recognized the change of testis in different groups involving the DM, BPH and the DM + BPH, the testes sizes in DM and BPH were reduced slightly, as contrast, EX has apparently recovered its weight and size (Fig. 2a-c).

Increased body weight and body mass index have been confirmed to be the risk factors of prostate enlargement [21–23]. The results presented here are controversial; we demonstrated that the body weight severely decreased, suggesting pathological changes in the STZ-induced DM, testosterone-induced BPH, and innate human DM models. Previously, we demonstrated that EX was able to suppress the prostatic inflammation in BPH [7]. A similar result regarding the DM group was reported by Belotto et al. [24].

### The pathological etiology of dyslipidemia of BPH is entirely different from DM

In Fig. 4a-b, we showed the dyslipidemic pattern without and/or with EX intervention in BPH rats was quite different from that of DM and BPH + DM rats. Although both dyslipidemic profiles were very alike, yet the DM patients usually show more severe hyperlipidemic manifestations than the BPH (Fig. 4a-b). Literature has indicated that metabolic syndrome-associated dyslipidemia to be the major determinants of prostatic inflammation [25] and overgrowth [1]. Amazingly, EX seemed to have further elevated the TG level in BPH to 140.4 ± 17.4 mg/dL, yet still falling within the normal range (26–145 mg/dL) (Fig. 4b).

According to Nandeesha et al. [26], in human T2DM the levels, total cholesterol, and LDL-cholesterol were significantly higher and HDL-cholesterol was significantly lower in BPH cases as compared to controls. Hammarsten et al. also indicated that there was a larger prostate gland in men with obesity (*p* < 0.0001), and low HDL-cholesterol levels (*p* = 0.0132) than in men without these conditions [27].

Nonetheless, in this investigation we found these two types of dyslipidemia in fact were totally different from each other (Fig. 4a-b), implicating the pathological etiology is completely discrete from each other between BPH and DM.

### EX reduced collagen deposition

BPH is usually implicated in detrusor muscle hypertrophy in the early phases of outflow obstruction and deposition of increasing amounts of extracellular matrix (e.g., collagen) [24, 28]. Moreover, DM also induces stromal remodeling and a thickening in the acinar basement membrane of the prostate, accompanied by an increase and disorganization of its proteoglycans, chondroitin sulfate, and collagen [29], as observed in our BPH- and DM-induced models (Fig. 5c).

The combined effect of DM + BPH + EX is still unclear. Previously, we proved EX to alleviate BPH [7]. In this work, we demonstrated that EX not only apparently alleviated the hyperplastic epithelial and stromal growth, inhibiting collagen deposition in BPH (Fig. 5c), but also reduced the collagen deposition in the DM groups. By contrast, the literature demonstrated that in DM, EX restores the DM-induced specific ultrastructural changes in cardiomyopathy, alleviating these symptoms toward non-DM phenotypes, particularly in the mitochondria and extracellular matrix proteins [30].

### In the DM and BPH + DM groups, EX substantially decreased the blood glucose and increased the insulin levels

Almeida et al. [31] pointed that body adiposity and glucose homeostasis improved with chronic physical exercise in Wistar male rat model. In addition, total insulin content was reduced in group acute trained, insulin secretion stimulated by glucose was reduced in trained groups (the aerobic trained and the acute trained) [31]. According to Almeida et al. [31], a possible modulating action on insulin secretion is probably related to the association of chronic adaptation with an acute response on cholinergic activity in pancreatic islets. Speculatively, the elevation of IGF and TG and variation of prostate weight by EX could be also dependent on such a phenomenon.

Literature has warranted also that blood glucose concentration is associated with the risk of prostate enlargement. Diabetic patients were more than twice as likely to have prostate enlargement compared with men without DM [13]. Patients with MetS exhibit a high annual prostate growth rate [21–23]. BPH patients with MetS exhibit substantially higher serum glucose levels than do BPH patients without MetS [32]. In subjects with MetS, the fasting glucose levels are usually remaining at higher levels than the normal. Documented data revealed that prostate size correlates positively with the fasting glucose level (r =0.186, *p* = 0.007), but not with BMI, testosterone, insulin level, or insulin resistance (each *p* > 0.05) [30]. Upon multiple adjusted linear regression analysis, prostate size correlated with PSA (*p* < 0.01) and increased fasting glucose levels (*p* =0.023) [33]. Worth noting, Kim et al. also indicated that in non-DM BPH patients with normal testosterone levels, fasting glucose level is an independent risk factor for prostate hyperplasia [33].

In addition, Nandeesha et al. indicated that insulin level in human was significantly associated with prostate size, in human BPH cases. And more importantly in T2DM patients, insulin has been shown to be an independent risk factor in the development of BPH [26].

Previously, Hammarsten et al. had indicated that there was a larger prostate gland in men with non-insulin-dependent diabetes mellitus (NIDDM) (*p* = 0.0058) and high insulin levels (*p* < 0.0001) than in men without these conditions [27].

Taken together, evidently, different animal model (rats vs. human) and different etiological events (Type 1 vs Type 2 DM) could reveal different pathological and biochemical outcomes. We showed in this Type 1 rat model for BPH alone EX did not affect the insilin level, but in case of BPH + DM, the insulin level was substantially reduced in the BPH + DM rats, and EX was seen to slighly ameliorate the insulin level (Fig. 6b), suggesting that although insulin is an independent risk factor in the development of BPH, EX could more or less modulate the pathological (cholinergic adaptation, [31]) condition associated with BPH + T1DM (Fig. 6b).

Moreover, EX seemed to have alleviated the testosterone level in BPH alone group to reach a level higher than the normal (Fig. 7a) (which could be due to the testosterone therapy), while the fasting glucose levels all maintained at extremely high levels (Fig. 6a), implicating the risk to aggravate BPH in the T1DM rats.

### EX upregulated the IGF and alleviated the MetS

The IGF-1, although highly suppressed in the normal control, was substantially upregulated by EX in the BPH, DM, and BPH + DM groups (Fig. 6c).

The IGF, another mitogen, and an antiapoptotic agent, binds to the insulin receptor/IGF receptor, and stimulates prostate growth [2]. IGFBP-3 seems to be a mulifunctional protein, which can potentate or inhibit IGF activity [34]. Increased IGF levels and IGF binding proteins (IGFBPs) in prostate tissue and blood are associated with an increased BPH risk, because they regulate the levels of circulating androgen and growth hormone [2].

Regarding the volume variation of the total prostate (TP) volume or transitional zone (TZ) volume, literature has demonstrated that higher PSA (*p* < 0.001), larger waist circumference (*p* < 0.001) and higher IGFBP-3 expression levels (*p* = 0.024) are independently associated with higher TZ volume [34].

The increase of IGF in prostate cancer has been widely studied. But little has been reported on the possible role of IGF in BPH [35]. However, reduced modulatory IGF binding protein levels do seem to be associated with increased BPH risk [35].

Could this be the cause that IGF was decreased in BPH model (Fig. 6c) compared to the control? We really still are not very clear about this.

### EX alleviated the BPH and BPH + DM by suppressing the DHT levels, conversely, EX alleviated DM by upregulating androgens

Recently, we demonstrated that EX alleviated BPH [7]. Biochemically, BPH is considered to be an imbalance between androgen and estrogen [36], an overexpression of stromal and epithelial growth factors, cytokines, and steroid hormones [37, 38]. In the BPH groups, the levels of testosterone, 5α-reductase, DHT, and AR were all substantially upregulated (Fig. 7a-e), resulting in substantially increased PSA levels (Fig. 7f), a phenomenon being consistent with the manifestations usually seen in the clinical treatment of BPH. EX alleviated the BPH and BPH + DM by suppressing the DHT levels (Fig. 7c). Subnormal testosterone levels inhibited regular prostate proliferation and differentiation [39].

The biological significance of enzyme like “5-α-reductase” definitely is far different from that of the ‘androgen receptor protein’. 5-α-Reductase is responsible for the transformation of testosterone into DHT, the latter activates the translocation of cytosolic AR into the nucleus. In order to differentiate these two biochemical functions, we performed the Western blotting of 5-α-reductase (Fig. 7b), and alternatively, we adopted immunohistochemical (IHC) stain to examine the nuclear translocation of AR (Fig. 7d).

At this stage, we purposely utilized the IHC stain to observe the AR with the goal not only aiming at its quantities, but also its in vivo distribution site. As can be expected, after homogenized the cytosolic and the nuclear AR’s would be homogeneously mixed together, and at this stage, even though you could identify a tremendous amount of AR by Western blot, you still could not differentiate the activated AR and its localization into nuclei. On the other hand, we skipped the ER, because ER in reality is not involved in such a biochemical transformation and translocation mechanism.

In the DM group, AR was substantially downregulated (Fig. 6d and e), which was in agreement with Gorbachinsky and Liu and Wang [33, 40]. Literature indicated that the prostatic cytosolic AR content was negatively correlated with the plasma glucose levels [41]. We showed EX alleviated DM by upregulating androgens and AR (Fig. 7d), and EX increased the testosterone levels in the DM + EX group (Fig. 7a)

EX alters the sex hormones and their receptors associated with the balance between apoptosis and cell proliferation in the ventral prostate [41]. EX increased the plasma corticosteroid, DHT, and testosterone levels in the DM groups (Fig. 7a-c), and possibly via these mechanism prevented the apoptosis of glandular epithelium [42] and protected the prostate from MetS-induced prostate hypoxia, fibrosis, and inflammation [43].

The manifestations exhibited by the BPH + DM groups could become even more complicated. No substantial differences in the testosterone levels between the BPH + DM control and the BPH + DM + EX were observed. To compare, the 5α-reductase was upregulated in BPH + DM (Fig. 6b), while the level of DHT was downregulated (Fig. 7c), which implicates the lowering of PSA as shown in Fig. 5f. As contrast, in the BPH group only, EX downregulated 5α-reductase (Fig. 7b) and consequently, DHT was substantially downregulated (Fig. 6c), leading to the lowering of PSA level (Fig. 7f).

Interestingly, EX seemed to exhibit a biphasic action model between BPH and DM. In the BPH only, EX suppressed the serum level of testosterone, 5α-reductase and DHT (Fig. 7a-c). In the DM patients EX elevated T, 5α-reductase and DHT (Fig. 7a-c). While in BPH + DM, although EX stimulated the activity of 5α-reductase, however EX suppressed the DHT without affecting the testosterone level (Fig. 7a-c).

As well known, high zinc level tends to inhibit the activity of 5α-reductase [44]. The middle socioeconomic DM patients exhibited lower serum zinc levels (1.05–4.8 mg/dL in males and 1.7–3.5 mg/mL in females) than the normal non-diabetic population [45]. As contrast, no significant decrease in plasma zinc between BPH and normal controls [46]. Thus in the BPH patients, the production of DHT via the action of 5α-reductase could be merely via the plasma substrate testosterone level controlled kinetics (Fig. 7a-c). Nonetheless, in BPH complicated with DM, the plasma zinc level may play a very important role [45]. As seen, the activity of 5α-reductase was de-repressed in the DM and BPH + DM controls compared to the normal subjects (Fig. 7b). Furthermore, EX tends to accelerate sweating rate. Some endurance runners had significantly lower serum zinc concentrations (<11.5 μmol/L) than did men who were not participating in chronic exercise [47]. Such tremendous zinc loss by sweating could cause further lowering of serum zinc level, resulting in highly de-repressed 5α-reductase (Fig. 7b, c).

### EX effectively alleviated the oxidative stress in BPH + DM by suppressing the serum TBARS and H_2_O_2_

TBARS level was significantly increased in type 2 DM with the duration of disease and development of complications [48]. Similarly we showed the serum TBARs level was highly raised in both the T1DM and the BPH + DM rats (Fig. 9). MetS raised the oxidative stress expressed as H_2_O_2_ and MDA despite in T1DM or T2DM [49]. BPH is always associated with oxidative stress [50]. DM more severely raised the serum TBARs and H_2_O_2_ levels (Figs. 8, 9). We demonstrated that EX ineffectively suppressed the serum TBARs level in T1DM (Fig. 9). Consistent with this, Laaksonen and Sen reported that the increased plasma TBARS in the diabetic men both at rest and after exercise [51]. As contrast, EX effectively suppressed the serum TBARs level in the BPH + DM when compared to the BPH + DM control (Fig. 9). To compare, EX effectively inhibited the production of serum H_2_O_2_ levels in both the DM and BPH + DM groups (Fig. 8). The strongly negative association between plasma TBARS and VO_2_ max suggests that good physical fitness may have a protective role against oxidative stress [52]. Results implicated that the oxidative stress associated with DM was more likely related to the lipid peroxidation, whereas that in BPH was more likely due to the elevation of serum H_2_O_2_ level.

### EX effectively alleviated the inflammation in BPH + DM by suppressing the prostate IL-6

Although in BPH, interleukin-6 (IL-6) was localized predominantly in basal cells of epithelia, IL-6 receptor was expressed in benign prostatic tissue in both epithelial and stromal cells [51], no IL-6 expression was detected in stromal cells on immunohistochemistry [53]. IL-6 is a pleiotropic cytokine that interacts with its receptor in prostate cells, thus regulating proliferative response and differentiation in prostates. The consequences of increased IL-6 expression could play a role as a mediator of acute phase reaction and as a pleiotrophic cytokine influencing antigen specific immune responses and inflammation, as well as a growth factor for prostate epithelial cells [54]. We showed prostatic IL-6 was raised in BPH control, while EX only slightly yet significantly suppressed its level (Fig. 10). as contrast, IL-6 was substantially raised in the DM and BPH + DM controls, in these two groups EX was seen effectively suppressed the elevation of IL-6. In particular, the level of IL-6 in BPH + DM was effectively alleviated to a level as that of control, underlying the promising effect of EX for amelioration of the inflammatory manifestation of BPH + DM, but to a lesser extent for the DM.

### Considerable production of NO ameliorated BPH, DM, and BPH + DM

EX highly stimulated the serum NO production (Fig. 11). Pathologically speaking, BPH is always characterized by hypoxia and chronic inflammation [19]. NO plays a crucial role in the autonomic innervations of all compartments of prostatic tissues. In obstructive BPH, the nitrinergic innervation is reduced compared to that in a normal prostate tissue [55], resulting in an enhancement of vasodilatation and blood flow, a promising strategy for treating BPH.

To date, interest in the NO pathway as a potential pharmacological target to treat male LUTS is increasing. Thus, given a potential role of the NO-pathway in the prostate and LUT, enhancing NO production can be a promising strategy to control the smooth muscle function in the human prostate [56].

To emphasize, metabolic syndrome (MetS) is a complex, highly prevalent disorder and a worldwide epidemic. In T2DM, central obesity, insulin resistance, dyslipidemia, and hypertension are the main components of MetS [57]. As contrast, T1DM is insulin-dependent. In our experiment, Streptozotocin induces insulitis and subsequent degeneration of the Langerhans islets beta cells, so the model was a mimic of diabetes type 1 as evidenced by the lowered level of insulin, which in fact was very similarly to the patients affected by DM type 1. While DM type 2 patients, as consequence of insulin resistance, usually show normal or higher level of insulin. Consequently our findings have elicited limitations to the extension of these biochemical findings in a human setting of patients, mostly affected by DM type 2.

Despite T2DM our T1DM, there is growing evidence of the association of MetS with the initiation and clinical progression of BPH and PCa, molecular mechanisms and effects on treatment efficacy remain unclear [57].

## Conclusion

EX can alleviate BPH, DM, and BPH + DM. EX provokes androgen remodeling and the specific expression of NO which may play an essential role in enhancing the effect of EX. Data from the peer-reviewed literature suggest an association of MetS with BPH and Pca in humans, although the evidence for a causal relationship remains missing. MetS, including TDM and T2DM, should be considered a new domain in basic and clinical research in patients with prostatic disorders. Further research is required to better understand the role of MetS in BPH and PCa. Even so, clinical urologists need to be cognizant of the effect that MetS has on urologic diseases, as well as on overall patient health. It is of certain that this model in reality has raised some unexpected results that could be haphazard and the consequence due to the small sample size and the complicate model rather than a real biological effect of BPH and/or DM and/or EX. Hence a further research is required to better understand the effects of EX on the oxidative inflammatory pathway.

## Abbreviations

5αRd: 5α-reductase
AR: Androgen receptor
BPH: Benign prostatic hyperplasia
BG: Blood glucose
BSA: Bovine serum albumin
ECL: Enhanced chemiluminescence
CHOL: Cholesterol
DM: Diabetes Mellitus
DAB: Diaminobenzidine
DHT: Dihydrotestosterone
EX: Exercise
HDL: High-density lipoprotein
IGFBPs: IGF-binding proteins
IGF: Insulin-like growth factor
IL-6: Interleukin-6
ip: intraperitoneal
LDL: Low-density lipoprotein
LUTS: Low urinary tract symptoms
MetS: Metabolic syndrome
NO: Nitric Oxide
PSA: Prostatic-specific antigen
STZ: Streptozotocin
Ts: Testosterone
TG: Triglyceride
TNF-*α*: Tumor necrosis factor-α
T2DM: Type 2 DM
T1DM: Type-1 diabetes mellitus
WSLD: Western sample loading dye
